# Comprehensive Investigation of Stoichiometry–Structure–Performance Relationships in Flexible Polyurethane Foams

**DOI:** 10.3390/polym14183813

**Published:** 2022-09-12

**Authors:** Adam Olszewski, Paulina Kosmela, Adam Piasecki, Wiktoria Żukowska, Mariusz Szczepański, Paweł Wojtasz, Mateusz Barczewski, Roman Barczewski, Aleksander Hejna

**Affiliations:** 1 Department of Polymer Technology, Gdańsk University of Technology, Narutowicza 11/12, 80-233 Gdańsk, Poland; 2 Institute of Materials Engineering, Poznan University of Technology, Jana Pawła II 24, 60-965 Poznan, Poland; 3 Institute of Materials Technology, Poznan University of Technology, Piotrowo 3, 61-138 Poznan, Poland; 4 Institute of Applied Mechanics, Poznan University of Technology, Jana Pawła II 24, 60-965 Poznan, Poland

**Keywords:** flexible polyurethane foams, isocyanate index, cellular structure, mechanical properties, thermal properties, sound absorption, thermal insulation

## Abstract

Polyurethane (PU) foams are versatile materials with a broad application range. Their performance is driven by the stoichiometry of polymerization reaction, which has been investigated in several works. However, the analysis was often limited only to selected properties and compared samples differing in apparent density, significantly influencing their performance. In the bigger picture, there is still a lack of comprehensive studies dealing with the stoichiometry impact on PU foams’ performance. Herein, flexible PU foams with a similar apparent density but differing in the isocyanate index (IIso) (from 0.80 to 1.20) were prepared. The stoichiometry–structure–performance relationships were investigated considering cellular and chemical structure, as well as the static and dynamic mechanical properties, thermal stability, thermal insulation, and acoustic performance. For IIso of 1.00, the biggest cell diameters of 274 µm were noted, which was 21–25% higher compared to 0.80 and 1.20 values. Increasing IIso reduced open cell content from 83.1 to 22.4%, which, combined with stiffening of structure (rise of modulus from 63 to 2787 kPa) resulting from crosslinking, limited the sound suppression ability around five times. On the other hand, it significantly strengthened the material, increasing tensile and compressive strength 4 and 13 times, respectively. Changes in the foams’ performance were also induced by the glass transition temperature shift from 6.1 to 31.7 °C, resulting from a greater extent of urethane groups’ generation and additional isocyanate reactions. Generally, the presented work provides important insights into preparing flexible PU foams and could be very useful for the future development of these materials.

## 1. Introduction

**Figure 1:**
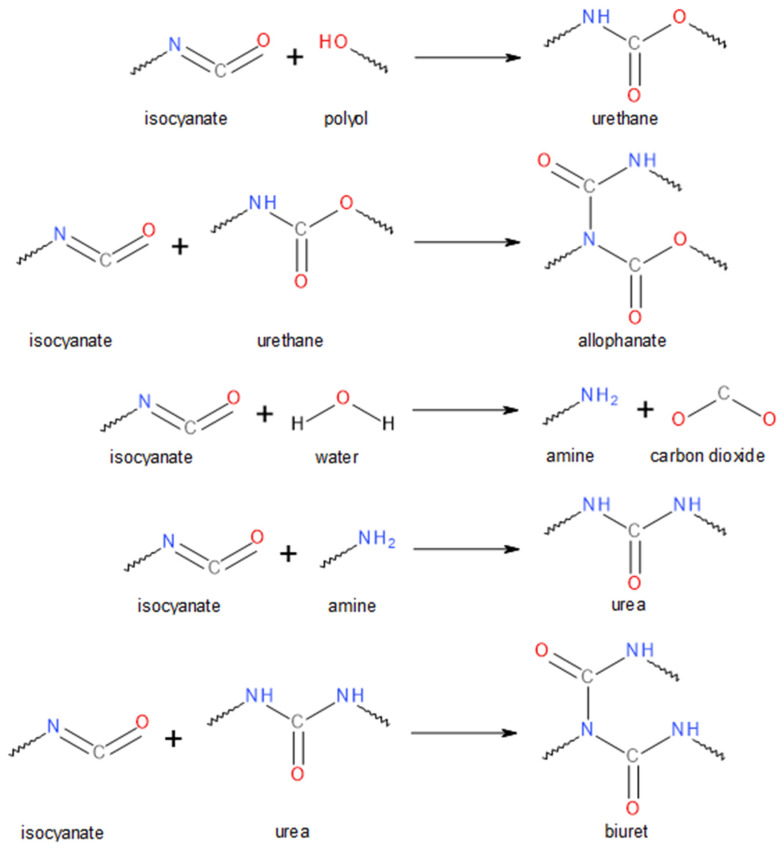
General scheme of isocyanate reactions during polyurethane foam manufacturing.

Polyurethane (PU) foams are versatile materials with a broad performance spectrum [1]. They account for around 50% of the global polyurethane market and are commonly applied in various industry branches depending on their actual structure and properties [2,3]. The most common applications of PU foams include bedding, furniture, building, construction, and automotive sectors [4,5,6]. The structure and resulting performance of polyurethane foams are directly impacted by their chemical composition, applied monomers, additives, and isocyanate index, which defines the ratio between the two most essential components considering the PU chemistry—polyols and isocyanates [7]. Precisely between the amount of hydroxyl and isocyanate groups present in the system prior to the polymerization [8]. These groups are essential for reactions leading to the generation of urethane bonds [9]. Moreover, except for the main reactions resulting in urethanes, isocyanates are taking part in other reactions leading to the formation of other bonds and groups crucial for polyurethanes’ structure and performance. The most important is a reaction between isocyanates and water, resulting in the generation of amine and carbon dioxide, which enables the foaming of the reaction mixture and the formation of foams’ cellular structure [10]. When isocyanates are present in excess in the system, so for values of isocyanate index exceeding unity, the unreacted isocyanate groups may further react with products of their previous reactions, so with urethane and amine groups. Such reactions yield allophanate, urea, and biuret groups, providing additional structure crosslinking [11]. Nevertheless, the biuret and allophanate linkages are thermos-reversible, so their impact on PU performance is temperature-dependent [12]. The general schemes of the isocyanate reactions are presented in Figure 1.

Typically, PU structures consist of two types of segments, where polyol macromolecules account for the soft segments, providing the material’s flexibility, while urethanes and other groups resulting from additional isocyanate-involving reactions are hard segments [13]. Therefore, rising values of the isocyanate index yield an increase in polyurethanes’ crosslinking, which affects the formation of cellular structure, as well as their overall performance, including mechanical, thermal, acoustic, and insulation properties [14,15].

The apparent density is another essential feature of polyurethane foams and all other cellular materials, which significantly affect their performance. It determines the actual amount of solid polyurethane in a given volume of material, showing the colossal impact on the mechanical, insulation, or acoustic properties [16,17]. Therefore, for a proper comparison, polyurethane foams should be characterized by a similar level of apparent density. Nevertheless, multiple works are not considering this parameter’s impact, which may yield misleading conclusions. Such an approach has been presented in multiple works dealing with the impact of the isocyanate index on the performance of polyurethane foams. Baghban et al. [18] investigated the influence of the isocyanate index on the acoustic damping ability of flexible PU foams. The authors indicated that the increasing share of isocyanates in the system enhanced the sound absorption coefficient. However, another effect of increased isocyanate index was a reduction of foams’ apparent density, which also has a very beneficial effect on sound absorption by facilitating sound dissipation as thermal energy [19]. Therefore, the direct contribution of the isocyanate index to the acoustic performance of foams cannot be determined. Similarly, the apparent density was not taken into account when discussing the influence of the isocyanate index on the mechanical performance of flexible polyurethane foams [20,21].

Moreover, even though several works evaluated the impact of the isocyanate index on the performance of flexible polyurethane foams [18,19,20,21,22], they are often limited to selected properties such as mechanical performance or acoustic damping. There is a lack of comprehensive studies investigating the performance of flexible PU foams from a broader perspective. Herein, in the presented study, we analyzed the consequences of the isocyanate index changes on the cellular and chemical structure and the performance of flexible polyurethane foams. The static and dynamic mechanical properties, as well as thermal stability, thermal insulation, and acoustic performance were analyzed, taking into account the influence of the isocyanate index on the size of cells share of open and closed cells. Moreover, contrary to the data reported in other research works, all analyzed samples were characterized by a similar apparent density, which is crucial for the performance of cellular materials. Such an approach enables a more straightforward analysis of obtained results because it eliminates the influence of one variable affecting foams’ properties and provides information about the actual impact of the isocyanate index.

## 2. Materials and Methods

### 2.1. Materials

Table 1 provides the information on the raw materials applied in the presented study.

### 2.2. Preparation of Flexible Polyurethane Foams

Polyurethane foams were manufactured on a laboratory scale by a single-step method. The values of the isocyanate index varied from 0.80 to 1.20. The raw materials were mechanically mixed for 10 s at 1800 rpm to ensure homogeneity of the reacting system. Subsequently, they were poured into a closed aluminum mold with dimensions of 20 × 10 × 4 cm. After demolding, the samples were conditioned at room temperature—21–23 °C and relative air humidity of 58–66% for 24 h. To provide a similar level of foams’ apparent density significantly affecting the performance of cellular materials, the amount of reaction mixture poured into the mold was adjusted. As a result, all foams were characterized by an apparent density of 187.5 ± 2.5 kg/m^3^. Table 2 provides the details of foam formulations.

### 2.3. Measurements

After conditioning, prepared PU samples were cut into specimens required for analysis following the standard procedures.

The Fourier transform infrared spectroscopy (FT-IR) analysis was conducted using a spectrometer Jasco FT/IR-4600 (Tokyo, Japan) at 23 °C in the Attenuated Total Reflectance (ATR-FT-IR) mode. A total of 32 scans at a resolution of 4 cm^−1^ were used to record the spectra of all analyzed samples.

The cellular structure of prepared PU foams was evaluated using a scanning electron microscope (SEM) MIRA3 from Tescan (Brno, Czech Republic). The JEE 4B vacuum evaporator from Jeol USA (Peabody, MA, USA) was applied to coat the analyzed foams with thin, approx. 20 nm carbon layer. The accelerating voltage of 5 kV was applied during analysis. The secondary electron detector was used.

The obtained images were analyzed with ImageJ software, and the following shape descriptors of cells were determined:

Circularity (C) calculated according to the following Equation (1):

(1) $$C=\frac{4\cdot A\cdot \pi }{P^{2}}$$

Aspect ratio (AR) calculated according to the following Equation (2):

(2) $$AR=\frac{L_{L}}{L_{S}}$$

Roundness (R) calculated according to the following Equation (3):

(3) $$R=\frac{4\cdot A}{\pi \cdot L_{L}^{2}}$$

where *P* is the perimeter; *L_L_* and *L_S_* are the lengths of the longer and shorter axis of the fitted ellipse, respectively; and *A* is the area of the fitted ellipse.

The Ultrapyc 5000 Foam gas pycnometer from Anton Paar (Graz, Austria) was applied to investigate the open cell content in prepared PU foams. Following measurement settings were applied: gas-nitrogen; target pressure—3.0 psi; measurement type-corrected; temperature control-on; target temperature—20.0 °C; flow mode-monolith; cell size—45 cm^3^; preparation mode—flow, 0.5 min. A gas pycnometer was also used to determine the density of solid portion of prepared foams. The following measurement settings were applied: gas—nitrogen; target pressure—10.0 psi; temperature control—on; target temperature—20.0 °C; flow mode—monolith; cell size—45 cm^3^; preparation mode—flow, 0.5 min.

The thermal conductivity coefficient (λ) of PU materials was determined with the heat flow meter Netzsch HFM 446 (Selb, Germany). The samples with dimensions of 20 × 10 × 4 cm were analyzed at the average temperature of 10 °C (1–19 °C range).

The determination of the sound absorption coefficients was conducted following the ISO 10534-2 [23] and ASTM E1050-8 [24] standards. The following equipment was used to carry out the tests: two BSWA impedance tubes (SW422 and SW 477), MC 3242 data acquisition hardware, PA50 power amplifier, BSWA VA LAB4 software (produced by BSWA-Technology Co. Ltd., Beijing, China), and two MI 19 microphones—¼ inch IEPE standard (produced by Roga Instruments, Germany). The measuring system was calibrated with a CA114 acoustic calibrator (BSWA Technologies Co. Ltd., Beijing, China). Atmospheric pressure, temperature, and air humidity were monitored with the LB-575 climate meter (produced by LAB-EL, Reguły, Poland). The preparation of samples for testing included cutting out from the base material (approx. 40 mm thick) and discs with a diameter of 30 and 100 mm. The samples were tested, from which about 8 mm of the uneven upper layer was removed. This treatment was also aimed at exposing the internal cellular structure of the material (foam). After cutting off the top layer, the samples were 32 mm thick.

The compressive strength of foams was estimated according to the ISO 604 standard using Zwick/Roell Z020 tensile tester (Ulm, Germany). The cylindric samples with dimensions of 20 × 20 mm (height and diameter) were applied. The test was conducted at a constant speed of 15%/min until reaching 60% deformation.

The tensile strength of foams was estimated according to the ISO 1798 standard using Zwick/Roell tensile tester (Ulm, Germany). The beam-shaped samples with 10 × 10 × 100 mm^3^ dimensions were analyzed using a constant speed of 500 mm/min.

Dynamical mechanical analysis (DMA) was carried out using a Q800 DMA instrument from TA Instruments (New Castle, USA). Cylindrical-shaped samples with dimensions of 10 × 12 mm were analyzed at the temperature range from −100 to 150 °C, with a heating rate of 4 °C/min.

The thermogravimetric (TGA) analysis was carried out using the Netzsch TG 209 F3 apparatus (Selb, Germany). Samples weighing approx. 10 mg were heated in a ceramic dish from 30 to 800 °C with a temperature increase rate of 10 °C/min under a nitrogen atmosphere [23,24].

## 3. Results and Discussion

**Figure 2:**
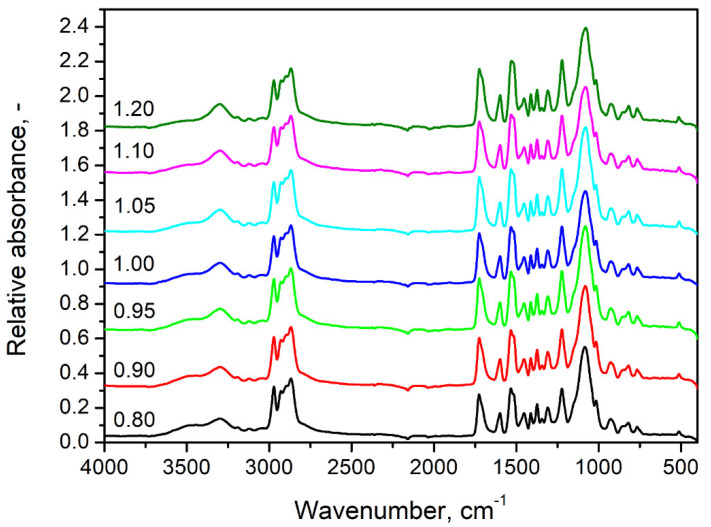
FTIR spectra obtained for prepared flexible PU foams with varying isocyanate indexes.

Figure 2 presents the FTIR spectra obtained for flexible PU foams with varying isocyanate indexes. It can be seen that all spectra show qualitatively similar appearances pointing to the presence of similar chemical groups in obtained materials. The presence of urethane bonds in all materials is confirmed by the signals related to the N-H, C=O, and C-N bonds [25,26]. Broad absorption bands at 3230–3370 cm^−1^ were attributed to stretching vibrations of N-H bonds, while peaks at 1515–1530 cm^−1^ were associated with the bending N-H vibrations [27]. Carbonyl bonds can be recognized by the presence of peaks characteristic of their stretching vibrations at 1705–1725 cm^−1^ [28]. Signals at 1220–1225 cm^−1^ are typical for stretching vibrations of C-N bonds [7]. The presence of the above-mentioned signals confirms the efficient generation of urethane bonds during PU foams’ preparation (see Figure 1).

Other notable absorption bands were noted at 2870–2970 cm^−1^ and 1010–1100 cm^−1^ and were attributed to the presence of C-H and C-O bonds, respectively. Their magnitude was hardly affected by the changes in the isocyanate index because they are not directly related to the generation of urethane bonds but rather to the chemical structure of particular foams’ components.

**Figure 3:**
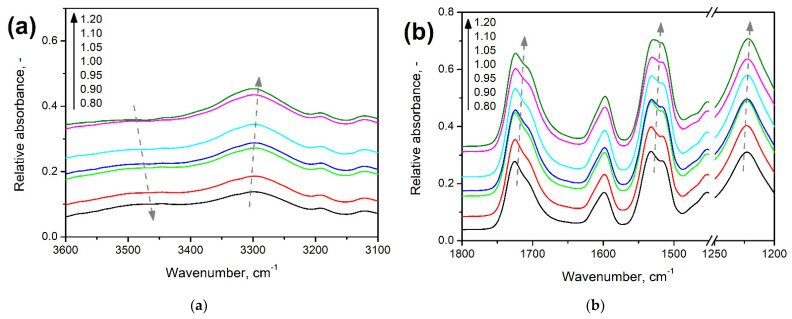
Detailed analysis of changes in FTIR spectra resulting from increasing isocyanate index at wavenumber range of (**a**) 3100–3600 cm^−1^ and (**b**) 1200–1800 cm^−1^.

Figure 3 points to the changes in the magnitude of particular peaks present in the foams’ FTIR spectra resulting from the increase in the isocyanate index. Figure 3a indicates the slight increase in the intensity of signal characteristics for vibrations of N-H bonds in urethane groups. On the other hand, above 3400 cm^−1^ can be observed broad and relatively weak band characteristics for stretching vibrations of hydroxyl groups are present in the structures of applied polyols [29]. Its magnitude is decreasing with the increase in the isocyanate index and, together with the strengthening of N-H signals, points to the efficient generation of urethane bonds in the reaction between hydroxyl groups of polyols and isocyanates. Nevertheless, these bands are typically broad and relatively weak, so the effect is insignificant [30].

Figure 3b visualizes the changes in FTIR spectra in the wavenumber range of 1200–1800 cm^−1^. It can be seen that the magnitude of all signals confirming the presence of urethane bonds is increasing with the isocyanate index. Moreover, the structure of bands associated with vibrations of C=O and N-H bonds is slightly changing due to the generation of shoulder bands, which points to hydrogen bonding [31]. Such an effect is typical for the increasing isocyanate index and has been observed by other researchers [32]. Considering the performance of prepared flexible PU foams, it can be beneficial for the mechanical properties due to the additional physical crosslinking [33,34,35].

**Figure 4:**
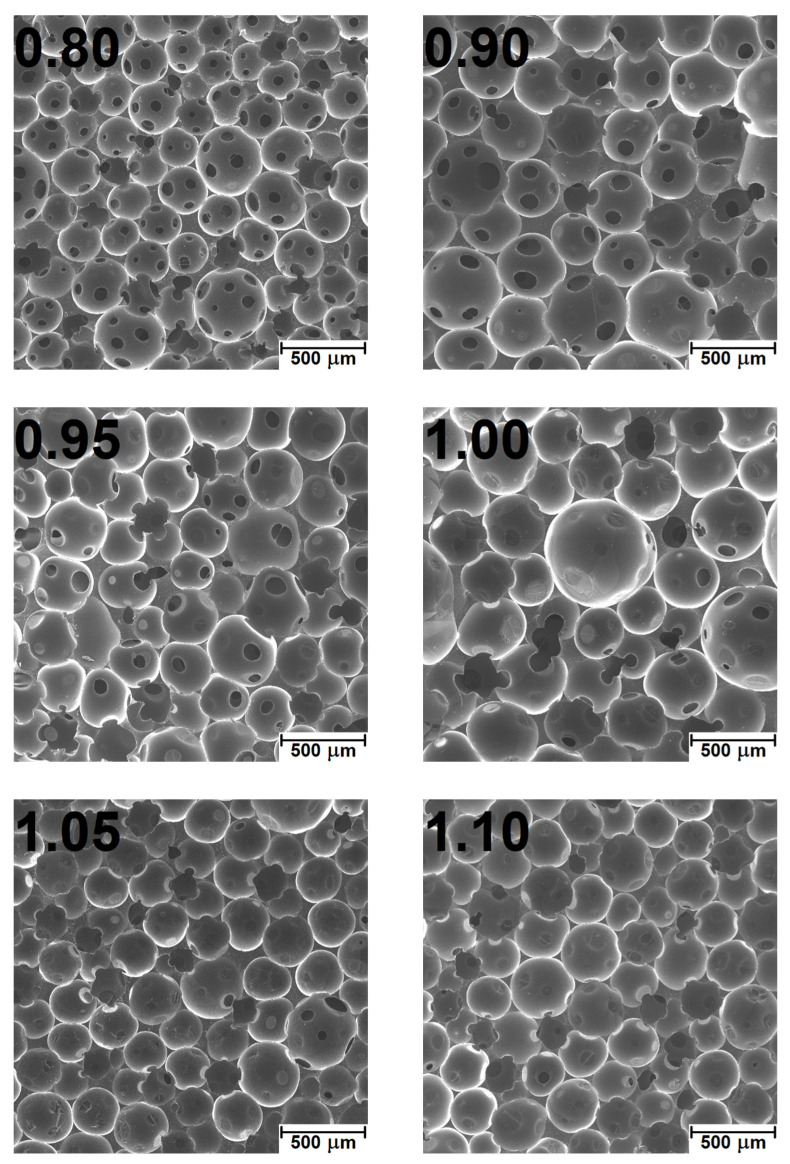

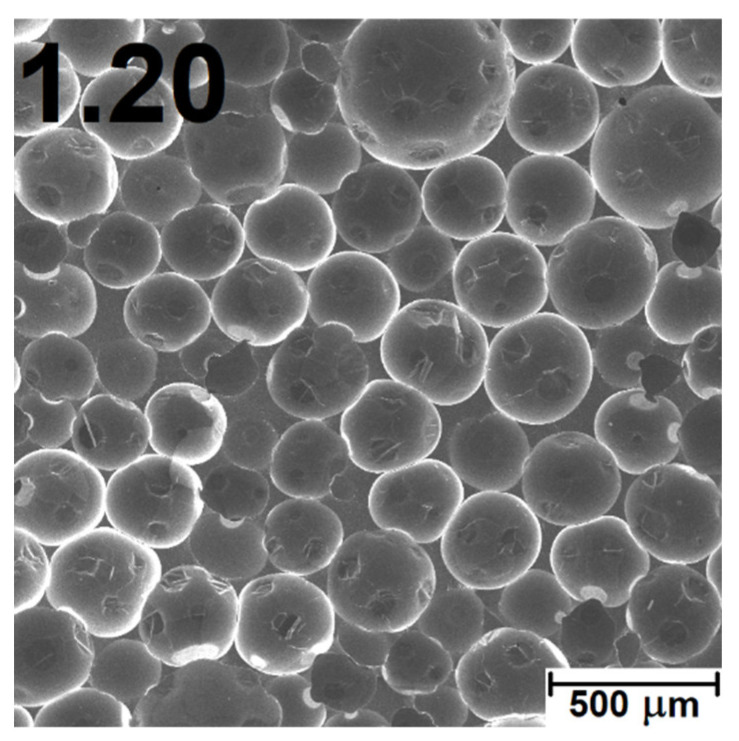
Images of prepared PU foams’ cellular structure obtained by SEM analysis.

Figure 4 presents the SEM images of foams’ cellular structure. The parameters describing the cellular structure determined during the images’ analysis are presented in Table 3. It can be seen that the biggest cells were obtained for the isocyanate index of 1.00. Such an effect could be associated with the changing balance between the strength of the cellular structure, degree of foams’ crosslinking, and amount of generated carbon dioxide. The initial increase for lower values of the isocyanate index can be associated with the increasing amount of carbon dioxide generated in the reactions between isocyanate groups and water used as a chemical blowing agent [36]. Moreover, with the increasing isocyanate index from 0.80 to 1.00, the strength of cell walls increases due to the stronger primary gel reactions and more rapid growth of reacting mixture viscosity, which results in the decreasing number of holes in cell walls [37]. Similar observations were made by Prociak et al. [22] and Bernardini et al. [20]. Therefore, with the increasing amount of CO_2_, the foaming cellular structure was able to keep more gas inside cells without the rupture of cell walls. Such an effect also resulted in the reduced content of open cells. With a further increase in the isocyanate index, the average cell diameter was reduced due to the excess of isocyanate groups in the system [38]. It enhanced crosslinking and strengthened the cellular structure by forming more urea and biuret groups in the reactions of isocyanates with amines and ureas, respectively. A stronger structure reduced the cells’ coalescence during foaming [39].

Table 3 also provides the values of foams’ apparent density and porosity, which were determined using Equation (4):

(4) $$p=1-\frac{\rho_{apparent}}{\rho_{solid}}$$

where *p* is the porosity, %; *ρ* is the density, kg/m^3^.

As mentioned in Section 2.2 Preparation of flexible polyurethane foams, all foams were characterized by a similar apparent density, which enabled their efficient comparison. Therefore, they also showed a similar level of porosity, which for all samples was in the range of 82.82–83.24%. Such an effect can be attributed to only minor, often negligible, changes in solid PU density with varying isocyanate index, as reported by other researchers [40,41].

Another essential factor of cellular materials related to their frequent applications is the thermal insulation performance. Even though flexible PU foams are hardly applied as thermal insulation materials, producers of different products from flexible foams used in the building industry provide the values of their thermal conductivity coefficients (λ) because they can contribute to the overall thermal characteristics of the building. However, this contribution is hardly significant due to the noticeably inferior thermal insulation performance compared to the typical insulation materials such as rigid PU foams or mineral wool [42]. Nevertheless, materials prepared in the presented study were also analyzed regarding their insulation performance. The impact of particular parameters of cellular structure on the thermal insulation performance of PU foams has been described in detail in our previous works [43,44]. Briefly, beneficial effects can be attributed to the drop in apparent density, reduced average cell diameter, and decreased open cell content. The apparent density quantifies the share of solid material in a given volume of foam, so it is critical for the contribution of solid and gas parts to the overall λ coefficient. Gases included in foam cells, even CO_2_ and air, are characterized by significantly lower values of λ coefficient than solid PU (even 15 times) [43]. Therefore, increasing apparent density results in the rise of the thermal conductivity coefficient. Considering cell diameter, its decrease reduces heat transfer through radiation, lowering the overall λ coefficient [45]. The content of open cells is more critical for the aging of thermal insulation materials because it affects the rate of gas exchange and substitution of applied blowing agents by air. Such a phenomenon plays a more critical role in rigid PU foams, where hydrofluorocarbon blowing agents are applied than in water-based foams, where cells are filled with carbon dioxide [46].

In the presented case, the λ values were relatively similar due to the slight differences in the most critical parameters—apparent density and average cell diameter—as reported in Table 3. Moreover, changes in these parameters were not proportional to the isocyanate index, so there is no proportionality between the isocyanate index and thermal conductivity coefficients.

**Figure 5:**
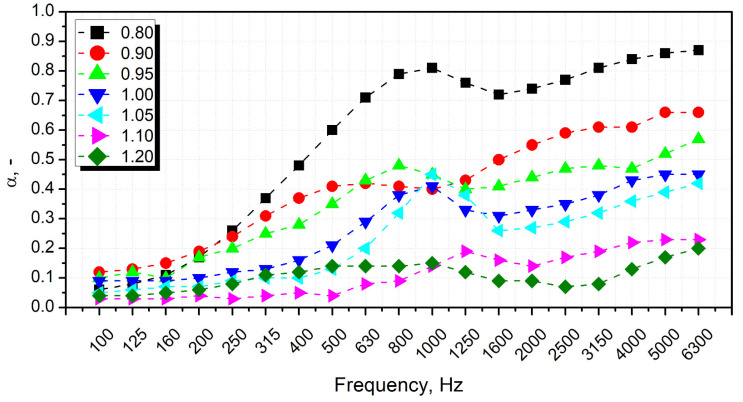
Sound absorption coefficient characteristics of PU foams with different NCO index in 1/3 octave bands (100–6300 Hz).

**Figure 6:**
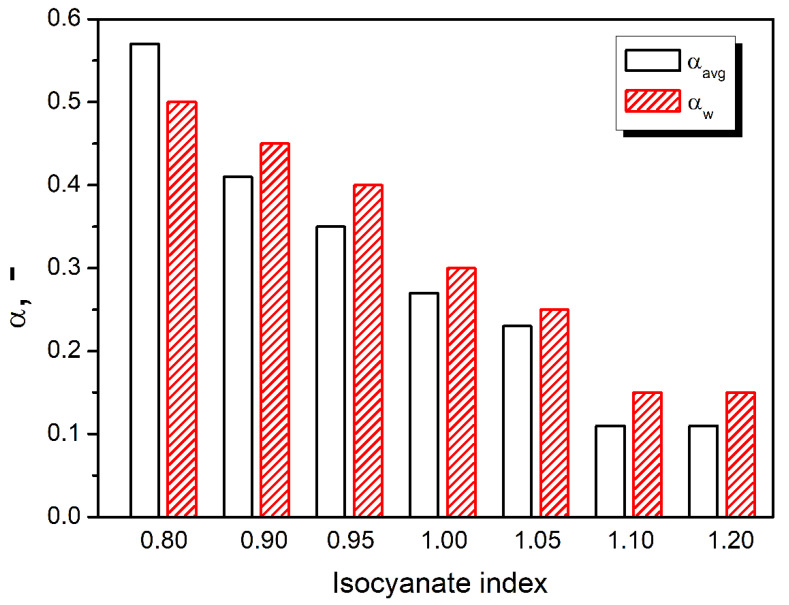
The average value of the sound absorption coefficient α_avg_ and the weighted sound absorption coefficients α_w_ of PU foams with different NCO index.

The results of the sound absorption test are presented in Figure 5 in the form of characteristics containing the values of sound absorption coefficients in 1/3 octave bands (100–6300 Hz). The characteristics were created based on partial results obtained in the bands 63–500 Hz and 250–1600 Hz (using an impedance tube SW422 with a diameter of 100 mm and different spacing of microphones) and in the band 1000–6300 Hz (using a tube SW477 with a diameter of 30 mm). The final result for each sample is the result of averaging three measurements. In addition, for each type of material (samples), the average value of the sound absorption coefficient α_avg_ and the weighted sound absorption coefficients α_avg_ were determined (Figure 6). Values were calculated according to Equation (5):

(5) $$\alpha_{avg}=\frac{1}{n\cdot \sum_{i=1}^{n}\alpha_{f\left(i\right)}}$$

where *α*_*f*(*i*)_ is the sound absorption coefficient (at the center frequencies *f*(*i*) from 100 Hz to 6.3 kHz) and *n* is the number of 1/3 octave bands.

The two key characteristics of porous materials predisposed for good sound attenuation should have an open-cell structure and pore size [47,48]. Prepared PU foams presented behavior typical for such materials, with sound absorption coefficient increasing with sound frequency [49]. Such an effect is associated with the decreasing wavelength and facilitates the penetration of PU cellular structure by sound waves. Foams showed good acoustic properties with an NCO index below 1.05. Significant differences between the series are already visible at frequencies above 200 Hz. Observing the SEM images showing structural changes in prepared foams, samples with a lower isocyanate index are characterized by a much higher content of closed pores, which was also confirmed by pycnometric analysis. Closed-cell structure has limited ability to absorb sound waves, which has been reported in literature works [17,50]. The foams revealed a similar size of pores. However, one should pay attention to the non-linear dependence of the decrease in the sound absorption coefficient in the 400–1250 Hz range, which does not occur with the increase in the isocyanate share. The more favorable acoustic properties of the 0.95 and 1.0 NCO/OH systems are probably due to the larger size of the pores in the structure.

The mechanism of sound suppression by porous systems is based on the phenomenon that sound waves penetrating the cellular structure promote vibrations of cell walls and the air inside them [51]. Damping of the induced vibrations by the cellular structure is attributed to the micro-deformations of walls and the friction between walls and airflow. Consequently, the sound energy is converted into heat [52]. Due to the greater deformation of cell walls noted for structures characterized by larger cell sizes, a more notable amount of sound waves’ energy is converted into kinetic energy, providing a more effective sound attenuation phenomenon [53]. Therefore, the efficiency of sound absorption will also include the flexibility of spurts [18]. Taking into account the results of mechanical tests, it can be concluded that the lower crosslink density and reduced stiffness translated into an increase in the dissipative effect expressed by the higher values of loss tangent for foams with isocyanate index below unity as revealed by thermomechanical properties’ analysis [54]. These results align with the acoustic studies’ results, in particular, the changes in averaged and weighted sound absorption coefficients. When the average sound absorption coefficient is higher than 0.2, the material can be considered a sound-absorbing material applied to the buildings’ insulation [55]. All tested materials revealed higher α_avg_ values in the frequency range of 100–6300 Hz, which allows classifying series with NCO index below 1.05 as materials with acoustic insulation potential [18,51,52,53,54,55].

**Figure 7:**
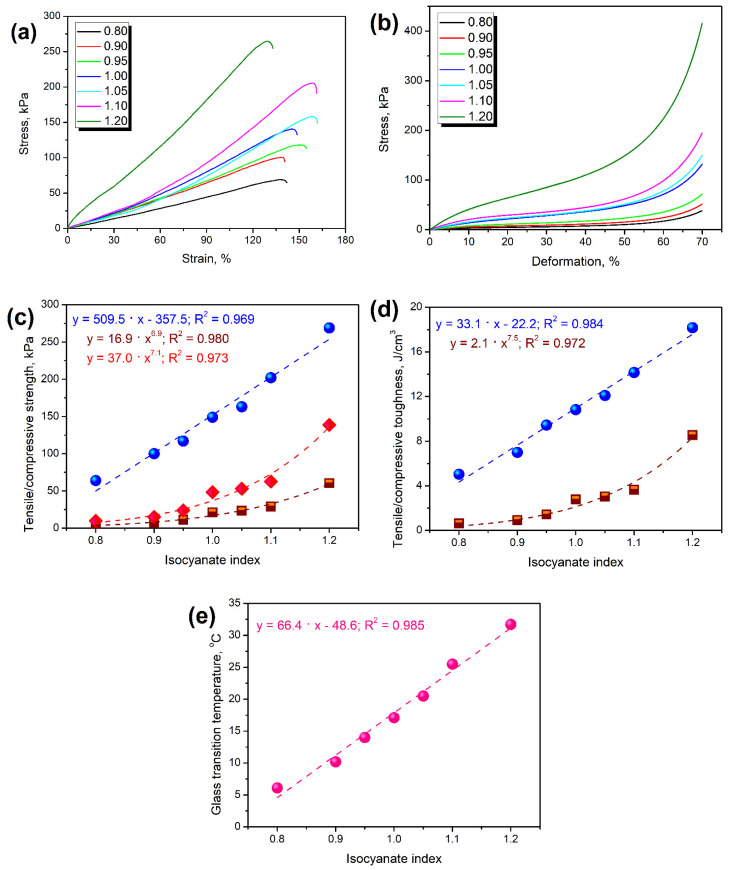
The influence of isocyanate index applied during foam preparation on (**a**) tensile stress–strain curves, (**b**) compression stress-deformation curves, (**c**) tensile (blue) and compressive (brown—at 20% deformation; red—at 50% deformation) strength, (**d**) tensile (blue) and compressive (brown) toughness, and (**e**) glass transition temperature.

Table 4 presents the parameters describing the mechanical performance of prepared flexible PU foams and their variations with changes in the isocyanate index. Figure 7 presents the impact of the isocyanate index on prepared foams’ tensile and compressive performance. Clearly, in the evaluated range, the positive correlation between the isocyanate index and foams’ strength and toughness can be noted. Additional data related to the regression carried out on data characterizing the mechanical performance of PU foams are presented in Tables S1–S6. Considering the elongation at break, the impact of IIso was noted only for the extreme values of 0.80, 0.90, and 1.20, when it did not exceed 140%. For IIso, between 0.95 and 1.10, the changes were negligible considering standard deviation, so they should be considered similar. Such an effect can be associated with the stoichiometric changes resulting from IIso variation. At lower values, the PU structure was not strong enough to withstand higher deformations, while at the highest value of 1.20, it was too stiff, so the macromolecular motions were restricted. For values close to the unity, the ratio between isocyanate and hydroxyl groups was balanced, so these two effects deteriorating foams’ ductility were weaker. For a proper understanding of the changes occurring in the materials’ mechanical performance, it is essential to refer to the glass transition temperature (T_g_), which in the case of polyurethanes, is very sensitive to the isocyanate index changes [41,56]. For the prepared materials, T_g_ was determined as the temperature position of a peak on the loss tangent (tan δ) temperature curve. As presented in Figure 7e, its values were directly proportional to the applied isocyanate index, which significantly influenced the mechanical properties of foams, which were determined at the ambient temperature, around 22–23 °C. The strength of foams was noticeably affected by applied formulation, which implicated a shift of T_g_. Both tensile and compressive performance changed with the isocyanate index. For tensile strength and toughness, the dependence was linear, while the compression power function was more suitable for describing the isocyanate index impact. Similar results were noted by Rojek and Prociak [37] and Prociak et al. [22], as well as in our previous work [57]. Such an effect is attributed to the above-mentioned reactions involving free isocyanate groups, which contribute to the increasing crosslinking of structure. The most gradual increase was noted for the highest values of isocyanate index when T_g_ noticeably exceeded ambient temperature.

Table 4 also provides information related to the dynamic mechanical performance of prepared foams. It can be clearly seen that the stiffness of foams, expressed by the storage modulus (E’), is notably affected by the applied formulation. A similar effect was observed in other works [21]. The isocyanate index quantifies the ratio between free isocyanate groups and hydroxyl groups present in the reacting PU system. The isocyanate index below unity indicates the deficiency of isocyanate groups yielding unreacted hydroxyls in the system [32]. Under conditions prevailing during polyurethane foaming, particularly at temperatures reached during volumetric expansion, these groups cannot react with themselves. Instead, they account for highly mobile loose chain ends, providing flexibility to the PU materials. As indicated in Figure 7e, it significantly affects the glass transition temperature, directly impacting the materials’ stiffness. For foams characterized by the T_g_ below 25 °C, the impact of T_g_ on the E’ is almost linear. Nevertheless, for foams, which are in a glassy state at 25 °C, a notable increase in stiffness is observed caused by the changes in the mobility of macromolecular chains [21,32].

Moreover, the values of *E*′, determined by DMA analysis, were used to calculate the brittleness of the foams, according to the Equation (6) presented by Brostow et al. [58]:

(6) $$B=\frac{1}{\varepsilon_{B}\cdot E\prime }$$

where *B* is the brittleness, 10^10^ %Pa; *ε_B_* is elongation at break, %; *E*′ is storage modulus at 25 °C, MPa.

From the above equation, it can be seen that stiff materials able to withstand a large range of deformation are characterized by low brittleness. Therefore, brittleness can be placed as the antagonist of toughness, often determined by the integration of stress–strain curves obtained during static mechanical tests. In their work, Brostow et al. [59] related these two parameters by developing the following Equation (7):

(7) $$\tau =\frac{b+c\cdot B}{1+a\cdot B}$$

where *τ* is the toughness, J/cm^3^; *a*, *b*, and *c* are constants.

Galeja et al. [60] described the brittleness–toughness relationship with a more affordable power function, enabling a more straightforward interpretation of the obtained results and proper understanding of input of particular constants, presenting the Equation (8):

(8) $$\tau =d\cdot B^{e}$$

where *d* and *e* are constants.

**Figure 8:**
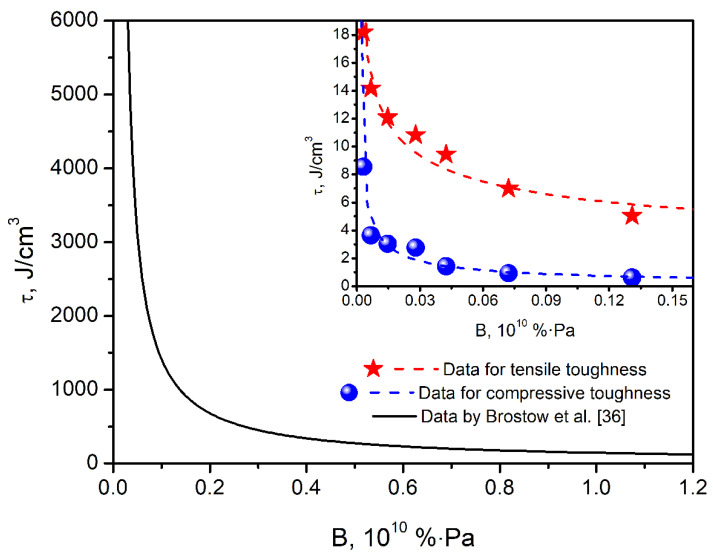
Brittleness vs. toughness plots for prepared PU foams compared with literature data by Brostow et al. [35].

Figure 8 shows the brittleness vs. toughness plots for prepared PU foams and for literature data by Brostow et al. [59]. Values of d and e constants are essential for data points lying on the curve bend at lower values of brittleness and toughness, which are characteristic of most polymeric materials. For data provided by Brostow et al. [59], who analyzed multiple homopolymers, copolymers, as well as steel and aluminum, values of d and e are 178.380 and −0.984, respectively. They did not analyze the foamed materials, whose mechanical performance is significantly different and related to their cellular structure. For analyzed samples, the values of d and e constants in the above equation were 3.086 and −0.316 for tensile performance and 0.185 and −0.651 for compressive performance. As a result, the curve characteristic for the data points developed in the presented work is characterized by the deeper bend lying closer to the coordinate system center, pointing to the inferior combination of composites’ toughness and brittleness compared to the literature data. Similar effects were noted in our previous works related to composite materials [61,62,63]. However, for composite materials, this effect was not as noticeable because it was attributed to the insufficient interfacial adhesion between matrix and filler, and the interface accounts only for the minor part of composite material. In the presented work, inferior mechanical performance was associated with the foams’ cellular structure and a significantly lower apparent density (in the range of 185–190 kg/m^3^) compared to the materials analyzed by Brostow et al. [59] (from around 910 kg/m^3^ for low-density polyethylene, through 1200 kg/m^3^ for polycarbonate, 1370 kg/m^3^ for polyethersulphone, 1780 kg/m^3^ for polyvinylidene fluoride, 2200 kg/m^3^ for polytetrafluoroethylene, 2700 kg/m^3^ for aluminum to around 7850 kg/m^3^ for steel). Therefore, future works dealing with the dependence between the materials’ toughness and brittleness should also consider the factor related to the material’s porosity.

**Figure 9:**
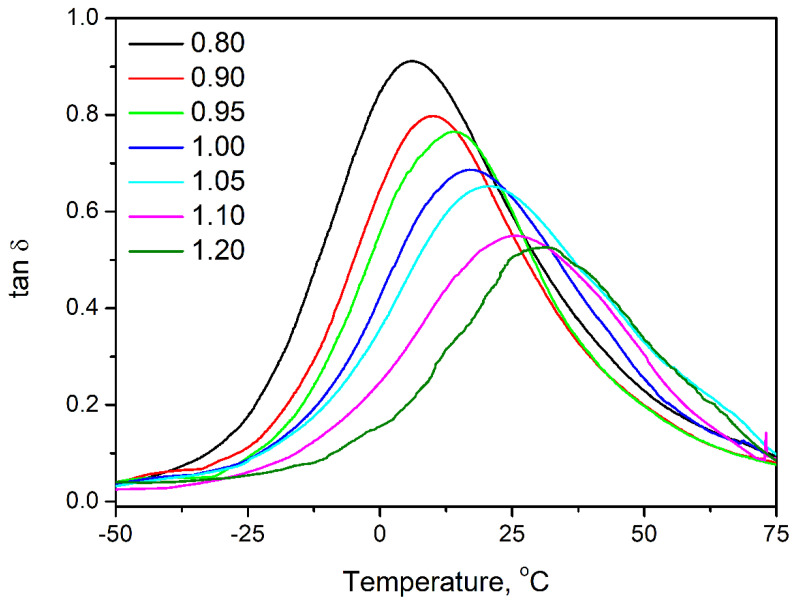
Temperature plots of tan δ for prepared flexible PU foams.

As mentioned above, the dynamic mechanical analysis also provides critical insights related to the materials’ loss tangent, often called the damping factor, as it quantitatively describes the ability to dissipate mechanical energy. Table 4, except for the values of T_g_, presents the values of tan δ at the glass transition temperature and area under tan δ peak determined from temperature curves presented in Figure 9. The magnitude of the tan δ peak is related to the materials’ ability to dissipate the energy through molecular motions [64]. The decreasing tan δ peak value suggests the reduced mobility of polymer macromolecules [61]. Herein, the magnitude of the tan δ peak, similar to its position indicating glass transition temperature, was strongly dependent on the isocyanate index. Its drop with the rising isocyanate index indicates the enhanced crosslinking of PU structure and the presence of more nodes in the macromolecular network due to the secondary reactions of isocyanates resulting in biuret and allophanate groups [61].

**Figure 10:**
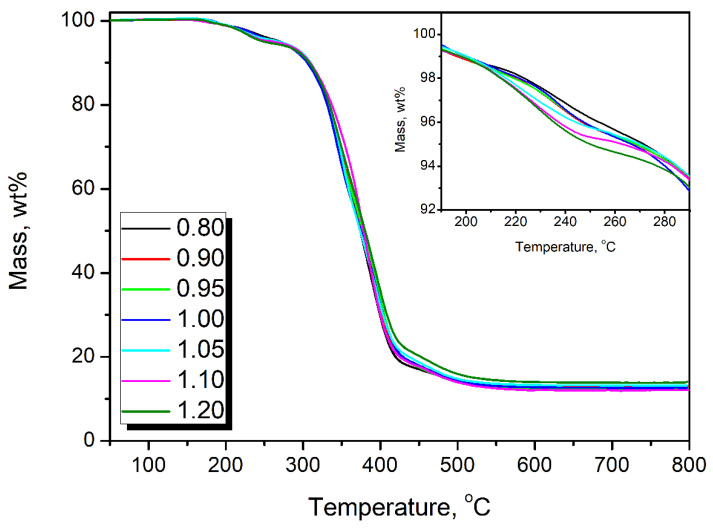
Mass loss curves showing the course of thermal decomposition of prepared flexible PU foams.

Figure 10 and Table 5 summarize the results obtained during thermogravimetric analysis of PU foams prepared with varying isocyanate indexes. It can be seen that all samples show a typical, two-step course of thermal decomposition related to the presence of two phases, urethane groups, and long macromolecules of applied polyols [65]. The first step, accounting for around 7–8 wt% mass loss, is attributed to the decomposition of hard segments, including mainly urethane groups, but also other groups enhancing the foams crosslinking, such as biurets and allophanates [66]. Due to the increasing amount of these groups, foams with higher isocyanate index were characterized by inferior thermal stability, which was expressed by the decrease in T_-2%_ and T_-5%_ parameters. For better visualization, Figure 10 includes the magnified thermogravimetric curves in the 190–290 °C temperature range when hard segment decomposition occurs [67].

## 4. Conclusions

The presented paper aimed to investigate the stoichiometry–structure–performance relationships in flexible polyurethane foams. Contrary to the data reported in other research works, all analyzed samples were characterized by a similar apparent density, which is crucial for the performance of cellular materials. The impact of stoichiometry was driven by varying the isocyanate index quantifying the molar ratio between isocyanate and hydroxyl groups in the reacting system. Flexible PU foams with isocyanate index from 0.8 to 1.2 were prepared and analyzed in terms of their cellular and chemical structure, as well as their static and dynamic mechanical properties, thermal stability, thermal insulation, and acoustic performance. The spectroscopic analysis confirmed the increasing extent of urethane group generation with the rise of the isocyanate index, as well as the potential generation of urethane groups indicated by the strengthening of N-H signals.

Stoichiometric changes also affected the cellular structure, whose impact is critical for the performance of foamed materials. The impact of IIso was not straightforward due to the contradictory influence on the strength of the cellular structure, degree of foams’ crosslinking, and amount of generated carbon dioxide affecting the extent of volumetric expansion. For values below unity, cell size increased with isocyanate index from 225 µm for 0.80 to 274 µm for 1.00, while further increase to 1.20 implicated decrease to 218 µm. Despite the intensified generation of carbon dioxide in the reaction between isocyanate and water, the open cell content decreased proportionally to the IIso increase from 83.1% to 22.4%, owing to the strengthening of cell walls resulting from stronger primary gel reactions and more rapid growth of reacting mixture viscosity. Changes in the cellular structure significantly affected the sound suppression ability of PU foams. Due to the closing of the cellular structure, the sound absorption coefficient dropped around five times. Foams showed good acoustic properties with an NCO index below 1.05. On the other hand, sound absorption ability was also affected by the mechanical performance of foams, particularly the damping factor, which quantifies the materials’ ability to dissipate energy. Strengthening of cellular structure and enhanced crosslinking implicated by increasing IIso noticeably reduced foams’ damping ability, which deteriorated sound suppression ability.

Considering mechanical performance, it was significantly enhanced by the increasing isocyanate index, which was related to the beneficial changes in cellular structure. Closing of cells and enhanced crosslinking caused the rise of tensile and compressive strength 4 and 13 times, respectively. Changes in stoichiometry induced stiffening of foams, gradually increasing storage modulus at 25 °C from 63 to 2787 kPa. Changes in the foams’ mechanical performance were also induced by the glass transition temperature shift from 6.1 to 31.7 °C, resulting from a greater extent of urethane groups’ generation and additional isocyanate reactions.

Changes in foams’ formulations and intensified isocyanate reactions also affected the thermal stability of materials due to the increased content of hard segments comprising of urethane groups, but also biurets and allophanates. They are characterized by inferior thermal stability compared to long macromolecules of applied polyols forming soft segments. As a result, the onset of thermal decomposition expressed by the temperature of 2 wt% mass loss was shifted from 223.5 °C for IIso of 0.80 to 214.0 °C for IIso of 1.20.

Summing up, the presented work provides important insights into preparing flexible PU foams by reporting results of a straightforward analysis of isocyanate index impact on PU foams’ performance without the influence of additional variables such as apparent density. Reported results could be very useful for the future development of foamed polyurethane materials.

## Figures and Tables

**Table 1:** Materials used to prepare flexible PU foams in the presented study.

Material	Producer	Properties/Additional Information
Rokopol^®^ F3000	PCC Group (Brzeg Dolny, Poland)	Polyether polyol, hydroxyl value—53–59 mg KOH/g
Rokopol^®^ V700	PCC Group (Brzeg Dolny, Poland)	Polyether polyol, hydroxyl value—225–250 mg KOH/g
Glycerol	Sigma Aldrich (Poznań, Poland)	Hydroxyl value—1800 mg KOH/g
SPECFLEX NF 434	M. B. Market Ltd. (Baniocha, Poland)	Polymeric methylenediphenyl-4,4′-diisocyanate, free isocyanate content—29.5%
PC CAT^®^ TKA30	Performance Chemicals (Belvedere, UK)	Potassium acetate catalyst
Dabco 33LV	Air Products (Allentown, USA)	Catalyst, 33 wt% solution of 1,4-diazabicyclo [2.2.2] octane in dipropylene glycol
Dibutyltin dilaurate	Sigma Aldrich (Poznań, Poland)	Organic tin catalyst
Distilled water	-	Chemical blowing agent

**Table 2:** Formulations of PU foams analyzed in the presented study.

**Component**	**Isocyanate Index**						
	**0.80**	**0.90**	**0.95**	**1.00**	**1.05**	**1.10**	**1.20**
	**Content, wt%**						
F3000	35.36	34.23	33.69	33.16	32.65	32.16	31.22
V700	35.36	34.23	33.69	33.16	32.65	32.16	31.22
Glycerol	0.85	0.82	0.81	0.80	0.79	0.78	0.75
DBTDL	0.64	0.62	0.62	0.60	0.59	0.58	0.56
33LV	0.42	0.41	0.41	0.40	0.39	0.39	0.38
TKA30	0.42	0.41	0.41	0.40	0.39	0.39	0.38
Water	0.36	0.35	0.35	0.34	0.34	0.33	0.32
pMDI	26.56	28.92	30.05	31.14	32.19	33.22	35.17

**Table 3:** Parameters describing the cellular structure and values of thermal conductivity coefficients for prepared PU foams.

**Parameter**	**Isocyanate Index**						
	**0.80**	**0.90**	**0.95**	**1.00**	**1.05**	**1.10**	**1.20**
Average particle size, mm	225 ± 85	252 ± 73	261 ± 79	274 ± 75	223 ± 62	226 ± 73	218 ± 65
Circularity	0.31 ± 0.13	0.30 ± 0.11	0.27 ± 0.13	0.31 ± 0.13	0.27 ± 0.10	0.31 ± 0.11	0.25 ± 0.08
Aspect ratio	1.30 ± 0.26	1.35 ± 0.29	1.34 ± 0.26	1.30 ± 0.24	1.30 ± 0.25	1.27 ± 0.24	1.25 ± 0.20
Roundness	0.80 ± 0.13	0.77 ± 0.14	0.77 ± 0.13	0.79 ± 0.13	0.80 ± 0.13	0.81 ± 0.13	0.82 ± 0.11
Open cell content, %	83.1 ± 3.6	74.3 ± 3.3	71.2 ± 3.2	59.9 ± 5.2	44.8 ± 1.5	33.3 ± 4.8	22.4 ± 2.5
Apparent density, kg/m^3^	185.3 ± 0.9	187.0 ± 2.0	188.8 ± 1.5	188.1 ± 0.9	188.1 ± 0.8	189.9 ± 1.6	186.5 ± 1.9
Porosity, %	83.13 ± 0.07	82.82 ± 0.24	82.99 ± 0.12	82.96 ± 0.07	83.01 ± 0.06	82.99 ± 0.21	83.24 ± 0.15
Thermal conductivity coefficient, mW/(m·K)	66.73 ± 0.84	66.80 ± 1.66	66.06 ± 1.10	67.42 ± 1.07	67.13 ± 0.80	70.12 ± 0.34	68.87 ± 0.54

**Table 4:** Mechanical properties of prepared flexible PU foams determined by static and dynamic mechanical tests.

		Isocyanate Index						
		0.80	0.90	0.95	1.00	1.05	1.10	1.20
Tensile strength, kPa		64 + 5	100 + 4	117 + 2	149 + 13	163 + 12	202 + 63	269 + 15
Elongation at break, %		138 + 6	140 + 1	154 + 13	159 + 15	160 + 12	159 + 6	131 + 2
Tensile toughness, J/cm^3^		5.036	6.993	9.437	10.823	12.101	14.156	18.172
Compressive strength, kPa	at 20% deformation	4.5 + 0.5	7.1 + 0.5	11.3 + 1.3	21.4 + 0.2	23.4 + 0.7	29.1 + 0.1	60.5 + 4.8
	at 50% deformation	9.9 + 0.9	15.0 + 0.2	24.0 + 2.7	48.4 + 1.1	53.1 + 2.9	62.5 + 1.2	138.9 + 13.7
Compressive toughness, J/cm^3^		0.628	0.927	1.436	2.774	3.033	3.637	8.549
E’ at 25 °C, kPa		63	114	187	270	502	1144	2787
Brittleness, 10^10^ %Pa		0.1309	0.0722	0.0425	0.0280	0.0147	0.0067	0.0030
T_g_, °C		6.1	10.2	14.0	17.1	20.5	25.5	31.7
tan δ at T_g_		0.912	0.799	0.766	0.689	0.653	0.555	0.532
Area under tan δ peak, °C		47.952	39.641	37.579	37.024	37.126	32.615	27.984

**Table 5:** Results of thermogravimetric analysis performed for prepared flexible PU foams.

Isocyanate Index	T_−2%_, °C	T_−5%_, °C	T_−10%_, °C	T_−50%_, °C	Char residue, wt%
0.80	223.5	271.4	309.2	373.8	12.51
0.90	219.9	267.9	308.8	377.0	13.07
0.95	219.2	268.2	308.9	378.2	12.18
1.00	221.6	266.6	305.5	374.9	12.62
1.05	216.1	270.1	309.9	373.8	13.21
1.10	214.4	262.8	309.2	378.3	12.11
1.20	214.0	250.0	308.1	380.3	14.12

## Data Availability

The data presented in this study are available in the Comprehensive investigation of stoichiometry–structure–performance relationships in flexible polyurethane foams.

## Supplementary Materials

The following supporting information can be downloaded at: https://www.mdpi.com/article/10.3390/polym14183813/s1, Table S1.The impact of isocyanate index on tensile strength – regression parameters and statistics.; Table S2.The impact of isocyanate index on compressive strength at 20% deformation – regression parameters and statistics.; Table S3.The impact of isocyanate index on compressive strength at 50% deformation – regression parameters and statistics.; Table S4.The impact of isocyanate index on tensile toughness – regression parameters and statistics.; Table S5.The impact of isocyanate index on compressive toughness – regression parameters and statistics.; Table S6.The impact of isocyanate index on glass transition temperature – regression parameters and statistics.
