# Supplementary material for: Comprehensive Investigation of Stoichiometry–Structure–Performance Relationships in Flexible Polyurethane Foams
Source: Polymers (Basel). 2022 Sep 12;14(18):3813. doi: 10.3390/polym14183813 (PMC9501594; doi:10.3390/polym14183813)
Supplement: Supplementary file 1 [file polymers-14-03813-s001.zip › polymers-1851979-supplementary.pdf]

Supplementary Materials

Comprehensive investigation of stoichiometry-structure-performance relationships in flexible polyurethane foams

Adam Olszewski [1], Paulina Kosmela [1], Adam Piasecki [2], Wiktoria Żukowska [1], Mariusz Szczepański [1], Paweł Wojtasz [1], Mateusz Barczewski [3], Roman Barczewski [4] and Aleksander Hejna [1,*]

[1] Department of Polymer Technology, Gdańsk University of Technology, Narutowicza 11/12 80-233 Gdańsk, Poland; adam.olszewski@pg.edu.pl (A.O), paulina.kosmela@pg.edu.pl (P.K.), s177319@student.pg.edu.pl (W.Ż.), s177460@student.pg.edu.pl (P.W.), s177276@student.pg.edu.pl (M.S.) , ohejna12@gmail.com (A.H.)
[2] Institute of Materials Engineering, Poznan University of Technology, Jana Pawła II 24, 60-965 Poznan, Poland; adam.piasecki@put.poznan.pl (A.P.)
[3] Institute of Materials Technology, Poznan University of Technology, Piotrowo 3, 61-138 Poznan, Poland; mateusz.barczewski@put.poznan.pl (M.B.)
[4] Institute of Applied Mechanics, Poznan University of Technology, Jana Pawła II 24, 60-965 Poznan, Poland; roman.barczewski@put.poznan.pl (R.B.)
* Correspondence: ohejna12@gmail.com

Table S1. The impact of isocyanate index on tensile strength – regression parameters and statistics.

Regression parameters			Statistics	
Regression type	linear		Residual Sum of Squares	732.48
Equation	$y = a \cdot x + b$		Total Sum of Squares	27992.00
Parameter	a	b	R^2 (COD)	0.974
Value	509.524	-357.524	Adjusted R^2	0.969
Standard Error	37.352	37.631	Residual Standard	12.10
Lower confidence limit (95%)	413.508	-454.258		
Upper confidence limit (95%)	605.540	-260.790		

Table S2. The impact of isocyanate index on compressive strength at 20% deformation – regression parameters and statistics.

Regression parameters			Statistics	
Regression type	non-linear		Residual Sum of Squares	36.02
Equation	$y = a \cdot x^b$		Total Sum of Squares	2176.17
Parameter	a	b	R^2 (COD)	0.983
Value	16.916	6.899	Adjusted R^2	0.980
Standard Error	1.269	0.495	Residual Standard	2.68
Lower confidence limit (95%)	13.654	5.627		
Upper confidence limit (95%)	20.179	8.171		

Table S3. The impact of isocyanate index on compressive strength at 50% deformation – regression parameters and statistics.

Regression parameters			Statistics	
Regression type	non-linear		Residual Sum of Squares	264.88
Equation	$y = a \cdot x^b$		Total Sum of Squares	11580.18
Parameter	a	b	R^2 (COD)	0.977
Value	36.979	7.147	Adjusted R^2	0.973

Standard Error	3.447	0.609	Residual Standard	7.28
Lower confidence limit (95%)	28.119	5.581		
Upper confidence limit (95%)	45.839	8.713		

Table S4. The impact of isocyanate index on tensile toughness – regression parameters and statistics.

Regression parameters			Statistics	
Regression type	linear		Residual Sum of Squares	1.58
Equation	$y = a \cdot x + b$		Total Sum of Squares	116.70
Parameter	a	b	R^2 (COD)	0.986
Value	33.111	-22.152	Adjusted R^2	0.984
Standard Error	1.735	1.748	Residual Standard	0.56
Lower confidence limit (95%)	28.652	-26.644		
Upper confidence limit (95%)	37.571	-17.659		

Table S5. The impact of isocyanate index on compressive toughness – regression parameters and statistics.

Regression parameters			Statistics	
Regression type	non-linear		Residual Sum of Squares	1.02
Equation	$y = a \cdot x^b$		Total Sum of Squares	43.62
Parameter	a	b	R^2 (COD)	0.977
Value	2.122	7.510	Adjusted R^2	0.973
Standard Error	0.214	0.650	Residual Standard	0.45
Lower confidence limit (95%)	1.572	5.838		
Upper confidence limit (95%)	2.671	9.182		

Table S6. The impact of isocyanate index on glass transition temperature – regression parameters and statistics.

Regression parameters			Statistics	
Regression type	linear		Residual Sum of Squares	6.00
Equation	$y = a \cdot x + b$		Total Sum of Squares	469.33
Parameter	a	b	R^2 (COD)	0.987
Value	66.429	-48.557	Adjusted R^2	0.985
Standard Error	3.380	3.404	Residual Standard	1.09
Lower confidence limit (95%)	57.742	-57.309		
Upper confidence limit (95%)	75.115	-39.806		
